# Lung cancer CT image generation from a free-form sketch using style-based pix2pix for data augmentation

**DOI:** 10.1038/s41598-022-16861-5

**Published:** 2022-07-27

**Authors:** Ryo Toda, Atsushi Teramoto, Masashi Kondo, Kazuyoshi Imaizumi, Kuniaki Saito, Hiroshi Fujita

**Affiliations:** 1 Graduate School of Health Sciences, Fujita Health University, Aichi, Japan; 2 Graduate School of Informatics, Nagoya University, Aichi, Japan; 3 School of Medicine, Fujita Health University, Aichi, Japan; 4 Faculty of Engineering, Gifu University, Gifu, Japan

**Keywords:** Lung cancer, Biomedical engineering

## Abstract

Artificial intelligence (AI) applications in medical imaging continue facing the difficulty in collecting and using large datasets. One method proposed for solving this problem is data augmentation using fictitious images generated by generative adversarial networks (GANs). However, applying a GAN as a data augmentation technique has not been explored, owing to the quality and diversity of the generated images. To promote such applications by generating diverse images, this study aims to generate free-form lesion images from tumor sketches using a pix2pix-based model, which is an image-to-image translation model derived from GAN. As pix2pix, which assumes one-to-one image generation, is unsuitable for data augmentation, we propose StylePix2pix, which is independently improved to allow one-to-many image generation. The proposed model introduces a mapping network and style blocks from StyleGAN. Image generation results based on 20 tumor sketches created by a physician demonstrated that the proposed method can reproduce tumors with complex shapes. Additionally, the one-to-many image generation of StylePix2pix suggests effectiveness in data-augmentation applications.

## Introduction

The remarkable development of artificial intelligence (AI) technologies, especially deep learning models, has led to the application of automated methods to medical images for a wide range of purposes, including lesion detection and segmentation and their classification as benign or malignant^1^. Typically, large amounts of training data are required to achieve good performances in these applications^2^. However, constructing large datasets of medical images is difficult owing to the need to protect patient information and collaboration among hospitals. Consequently, in some cases, performing AI methods remains insufficient. Hence, data augmentation has been performed using various methods^3^, typically image processing (e.g., rotation and flipping of images by geometric transformation, noise addition, and contrast modulation). However, the amount of data that can be increased using this method is limited. Recently, researchers have aimed to augment data by generating synthetic lesion images using generative adversarial networks (GANs) as an AI-based image-generation technology^4–8^. Moreover, a wide range of target modalities and tasks have been explored. For example, Waheed et al. performed data augmentation with a GAN model for a COVID-19 classification model using chest X-ray images^7^. Sandfort et al. used GAN-augmented data to perform multiorgan segmentation using computed tomography (CT) images^8^. These approaches have attracted attention as anonymization methods as they can generate new data without patient information^9^. Generally, GAN-based image-generation methods have prioritized the generation of a large number of images and have not sufficiently ensured the quality and diversity of the generated images^10^. Therefore, insufficient progress has been made in the application of GANs and AI to rare diseases where data augmentation is particularly important. Accelerating the application of AI to such diseases is possible by improving the diversity of the images generated by GANs. This study aims to apply a GAN model to generate lesion images of the desired shapes. In our previous study, we attempted to generate lesion images using InfoGAN^11^, which provides additional parameters for shape control^12^. However, this method requires manually adjusting several parameters for complex shape representation. Therefore, in this study, we propose using sketches as a more intuitive and convenient shape control method and generating images using pix2pix, an image-to-image translation technology based on a GAN model. In this study, as an initial investigation, the feasibility of the proposed method was verified using computed tomography (CT) images of lung cancer. Furthermore, a modified pix2pix named StylePix2pix was proposed. This model includes the following advantages: (1) lesion shape can be controlled using sketches, (2) one-to-many image generation can be performed, and (3) image generation can be conducted using the same procedures as for the original pix2pix (i.e., without requiring additional procedures).

## Related works

GANs are commonly applied as deep-learning image-generation techniques. Competitive training is performed by two different deep learning models, including a generator *G* that generates a fake image *G(z)* based on random numbers *z*, and a discriminator *D* that aims to correctly distinguish real images *x* from fake images *G(z)*, which represents the real image space *p*_data_ using the fake image space *p*_z_ to generate high-quality images. The loss function of this training can be formulated as a minimax game (Eq. (1)).

1 $$\begin{array}{c}\underset{G}{\text{min}}\, \underset{D}{\text{max}}V\left(D,G\right)={\mathbb{E}}_{x\sim {p}_{\text{data}}\left(x\right)}\left[\text{log}D\left(x\right)\right]+{\mathbb{E}}_{z\sim {p}_{z}\left(z\right)}\left[\text{log}\left(1-D\left(G\left(z\right)\right)\right)\right].\end{array}$$

Although no particular deep learning model must be applied to both the generator and discriminator comprising the GAN, deep convolutional GANs (DCGANs)^13^, which employ convolutional neural networks (CNNs), are widely used.

The images generated by regular GAN models depend on random input numbers and are difficult to control. Therefore, conditional GAN (cGAN) models were proposed to generate images for various classes by simultaneously inputting numerical information as labels to indicate the information of the subject^14^. Because real and fake images are conditioned by label *y*, the loss function of cGAN models may be formulated as given in Eq. (2).

2 $$\begin{array}{c}\underset{G}{\text{min}}\,\underset{D}{\text{max}}{V}_{\text{cGAN}}\left(D,G\right)={\mathbb{E}}_{x\sim {p}_{\text{data}}\left(x\right)}\left[\text{log}D\left(x|y\right)\right]+{\mathbb{E}}_{z\sim {p}_{z}\left(z\right)}\left[\text{log}\left(1-D\left(G\left(z|y\right)\right)\right)\right].\end{array}$$

Pix2pix, the model used in this study, was inspired by cGAN and enables image-to-image translation by adopting a U-net architecture for the generator and changing label *y* from simple numerical information to conditioning images^15,16^. Images before and after the translation must be paired with some meaning, such as black-and-white and color photographs of the same subject, or a map and an aerial photograph of the same location. The loss function of pix2pix is given by Eq. (3), and a regularization term using the L1 norm between the real and generated images is added to Eq. (2). This regularization is useful for improving image quality in image translation tasks^17^.

3 $$\begin{array}{c}{V}_{\text{Pix}2\text{pix}}=\underset{G}{\text{min}}\,\underset{D}{\text{max}}{V}_{\text{cGAN}}\left(D,G\right)+\lambda {\mathbb{E}}_{x,y,z}\left[{\Vert x-G\left(z|y\right)\Vert }_{1}\right].\end{array}$$

Image quality enhancement and translation between different modalities are main applications of pix2pix in medical images. A substantial number of versions have been developed with their own modifications, depending on their purpose and modality^18,19^. As an example of using sketch-like images together, Zhang et al. proposed SkrGAN, which used medical images such as chest X-rays and fundus images, and detected their edges via a Sobel filter to improve the representation of microstructures and blood vessels in image generation^20^. This is just a case of using sketches to improve the quality of generated images, which is essentially different from the approach taken in this study. Similar examples to our approach can be found in the following. Liang et al. used sketches as an aid to generate ultrasound images based on real images with new structures added^21^. For pulmonary nodules, Wang et al. proposed a method for guiding images generated using a mask of the surrounding area extracted from the real CT image and a free-form lesion mask^22^. Many of these cases require a base real image for image generation, and, while sophisticated image generation is possible, it is potentially limited by base real image. While generating completely new images is possible when only sketches are used as input for pix2pix, generating multiple images with different color tones and backgrounds using a single sketch is difficult with pix2pix as the generated image has a one-to-one relationship with the input image. Therefore, number of sketches must match number of images. Data augmentation by GAN typically requires generation of several thousand images, or at least of several hundred. However, an enormous amount of time is required to create sketches for all of these by hand. To address this, we propose StylePix2pix as an improved model of pix2pix based on the idea of StyleGAN in this study^23,24^. StyleGAN is an unsupervised GAN model developed to represent image features as styles, a concept known as style transfer^25,26^. Our StylePix2pix is designed to achieve diverse image generation by controlling tumor shapes using sketches and large-scale image generation sufficient for data augmentation applications.

## Methods

### Overview

**Figure 1:**
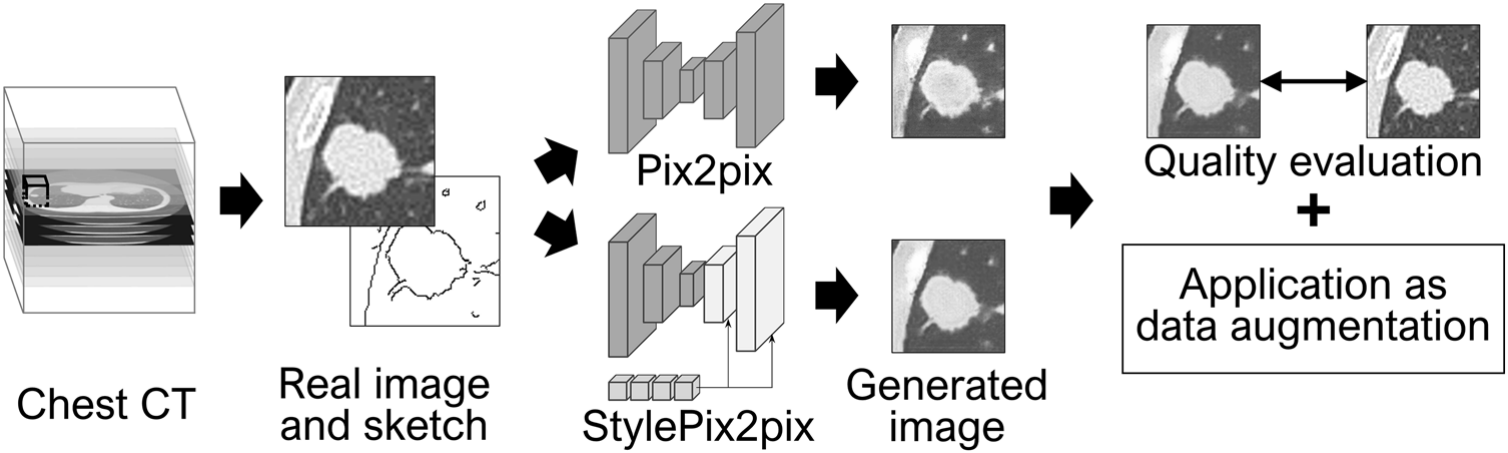
Outline of this study.

Figure 1 presents an outline of this study. Two-dimensional images of only lesions and their surroundings were obtained from chest CT images. The model was trained to generate images using recorded images and sketches created based on them. To verify the effectiveness of the proposed StylePix2pix model, we also generated images using the standard pix2pix model (or, simply, pix2pix) alone. To investigate the quality of the generated images, comparisons with real images were performed using both qualitative and quantitative evaluations. Additionally, the generated images were subjected to data augmentation for a classification task as a more practical evaluation.

### Image generation

#### Dataset details

We analyzed approximately 147 tumors in the chest CT images of 133 lung cancer patients collected at Fujita Health University Hospital. Of these, 20 tumors were used as the testing dataset, and the remaining 127 tumors were used for training. All images were acquired using an Aquilion ONE scanner manufactured by Canon Medical Systems and reconstructed by AIDR3D, using FC51 and FC52 as kernels for the lung window. The number of matrices was 512 × 512, pixel size was 0.625 × 0.625 mm^2^, and slice thickness was 0.5 mm. Data used in this study was approved by the Research Ethics Committee of Fujita Health University (No. HM17-002). All procedures were conducted in accordance with the Ethical Guidelines for Medical and Biological Research Involving Human Subjects in Japan, and informed consent was obtained from all participants.

#### Image preprocessing

From each CT image, the imaged tumor and its surroundings were cropped in cubes (representing volumes of interest (VOIs)) twice the size of the tumor’s long diameter. Crop size was determined to consider information on the surrounding structures in addition to the lesion, and similar settings or ideas have been used in various studies on pulmonary nodules^27–29^. As pixel size and slice thickness differed, each voxel was transformed into an isotropic cube with 0.625 mm on each side. As our models were designed for 2D images, from the cropped VOI, multiple 2D images were acquired for input to pix2pix and StylePix2pix. From all VOIs (training and testing data), an axial plane of the tumor center was obtained as a representative image of each VOI. For training data only, angled slices were additionally acquired at 10° intervals from the axial plane to ± 30° in the left–right and head–tail directions, respectively, to increase amount of data. All 2D images were resized to 128 × 128 pixels by linear interpolation and saved in a 24-bit (RGB) PNG format with the window level set to -600 and the window width set to 1600 as the lung window. The total number of 2D images in each dataset was 12,446 for the training dataset and 20 for the testing dataset.

Sketches must be created for all acquired 2D images. However, creating sketches for all images by hand was impractical; therefore, edges were detected by the Canny edge detection operator^30^ to substitute for sketches. The Canny method consists of four steps: (1) noise suppression using a Gaussian filter, (2) edge pre-detection using Sobel filters, (3) non-maximum suppression in the normal direction of each edge, and (4) edge determination using hysteresis thresholding. We used a 5 × 5 Gaussian filter in (1), an upper limit of 200, and a lower limit of 128 as the thresholds in (4). Edges were saved in an 8-bit (grayscale) PNG format.

#### Pix2pix

**Figure 2:**
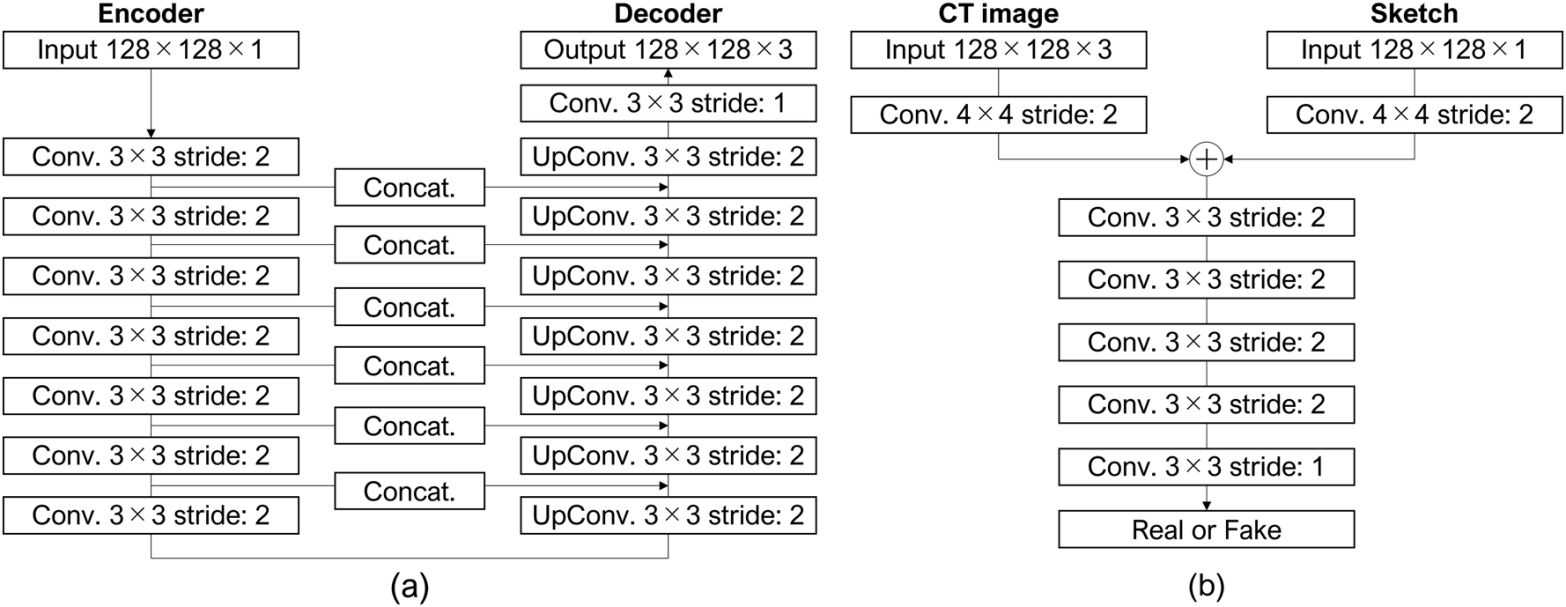
Network structure of pix2pix used in this study: (**a**) generator and (**b**) discriminator.

Figure 2 presents the proposed pix2pix model. It consists of a generator with a U-Net structure that performs seven down-sampling and up-sampling operations and discriminator using a simple CNN. Both sketches and CT images (real or generated) were inputted into the discriminator. The network was constructed and trained using Tensorflow2 on a server equipped with an NVIDIA Quadro RTX8000 GPU using Adam (β_1_ = 0.9, β_2_ = 0.999) as the optimization algorithm, with a learning rate of 1 × 10^–5^, 400 training epochs, and a batch size of 5.

#### StylePix2pix

**Figure 3:**
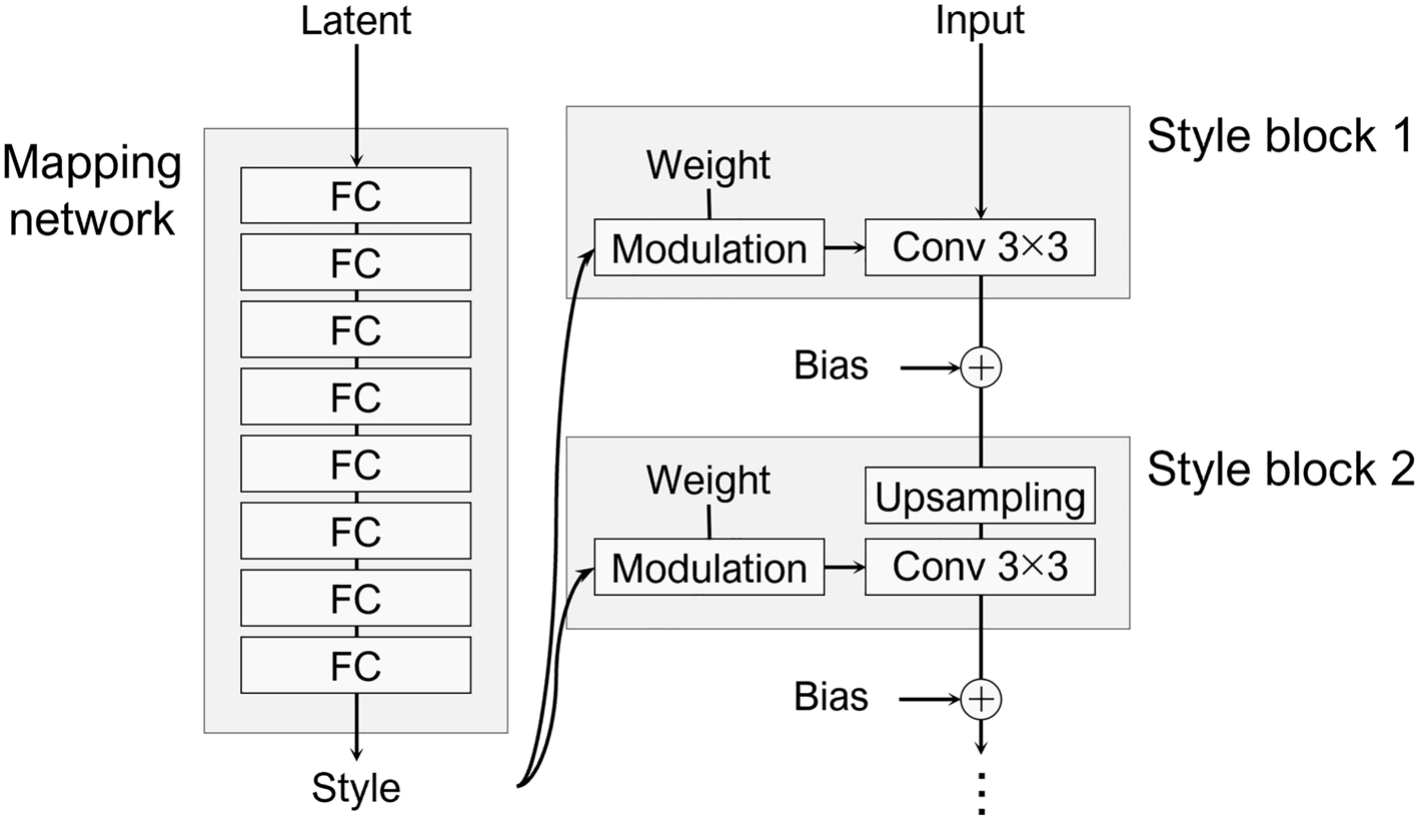
Basic structure of StyleGAN.

The original StyleGAN has two essential elements: a mapping network that generates style information using multiple fully connected layers (Fig. 3), and a mechanism called a style block that linearly transforms the kernel of each convolutional layer of the generator according to the generated style. The input (latent) of the mapping network consisted of random numbers. Diverse styles can be expressed without supervision, and various styles can be applied by adjusting input data. However, these operations do not involve directly inputting a style image, unlike the approach adopted for style transfer tasks^26^. Our StylePix2pix model introduces these mechanisms into pix2pix to examine whether they are also effective in pix2pix. As the nature of pix2pix and the original StyleGAN are different, experiments merging them are considered novel.

**Figure 4:**
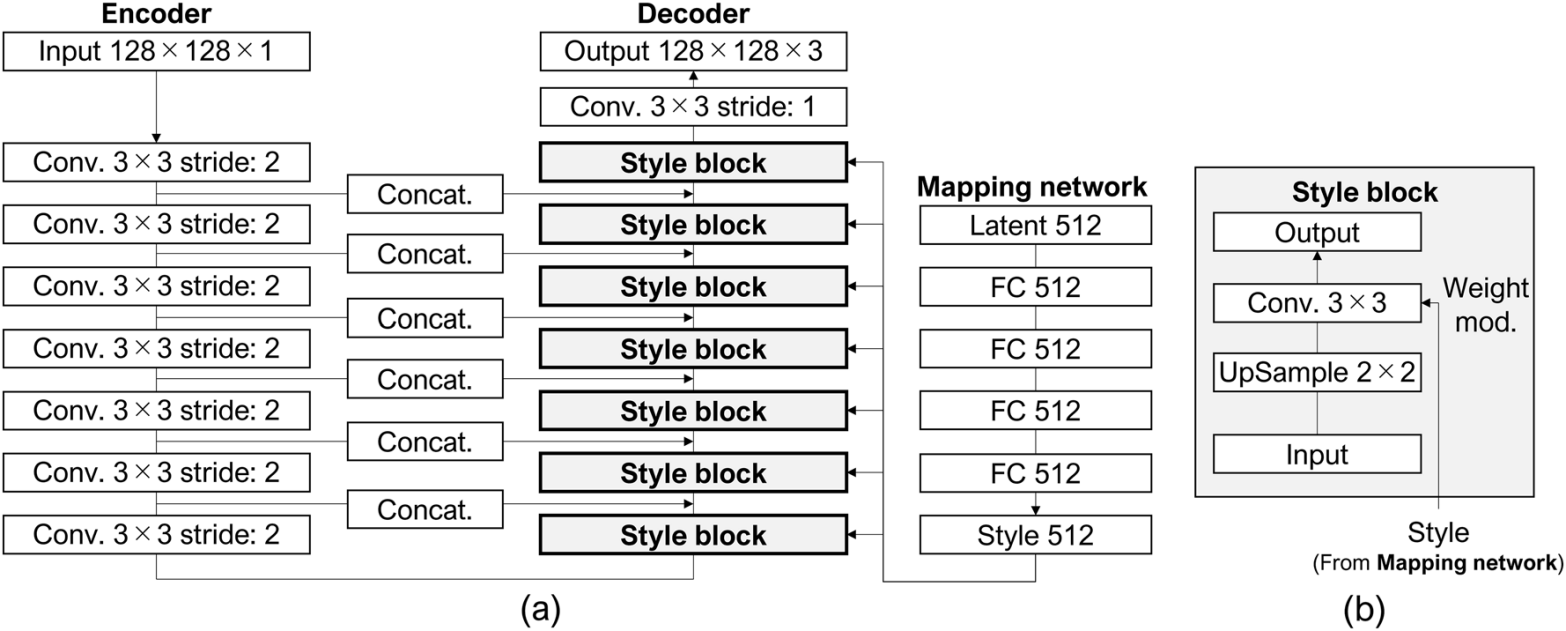
Network architecture of StylePix2pix. (**a**) Generator with style blocks and mapping network. (**b**) Structure inside the style block.

Figure 4 indicates that the structure of StylePix2pix retains the basic structure of the baseline pix2pix, adds a mapping network, and replaces all seven up-convolution layers of the generator with style blocks. The discriminator was unchanged from the standard pix2pix model. In each style block, the style is represented by four fully connected layers based on a 512-dimensional input, and the weights of the 3 × 3 kernel of the convolutional layer were adjusted based on style. The network was constructed, and hyperparameters were established in the same environment as pix2pix; however, we changed the number of training epochs to 200.

### Evaluation methods

#### Sketching by doctors

Although edges were used to train the model, we assumed the use of hand-drawn sketches. Therefore, we verified that images of sufficient quality can be obtained when sketches were used. Four doctors from the Department of Respiratory Medicine at Fujita Health University Hospital created sketches based on the CT images of the test data. To verify whether generating images from sketches of various patterns was possible, only the lesion was specified as the target to be drawn, and the accuracy of its shape, size, and position was not considered. No restrictions were placed on whether blood vessels and bronchi within the lung field were drawn as structures within the mediastinum and chest wall. This strategy is based on the idea that generating realistic nodule images from detailed sketches is possible. However, if generating images with a certain reality even from rough sketches is possible, the availability for data augmentation can be increased. Sketches were drawn on paper, digitized with a scanner, resized to 128 × 128 pixels, and binarized for use as data. During binarization, the threshold was adjusted for each image using visual feedback to avoid missing relevant information in the drawing.

#### Image quality metrics

Standard evaluation methods have yet to be established for images generated using GAN models^31,32^. We adopted two types of image quality metrics: a general image quality metric and an image similarity metric based on features extracted by a CNN. The peak signal-to-noise ratio (PSNR) and structural similarity (SSIM)^33^ were used for the former. Conversely, the Fréchet inception distance (FID)^34^ and learned perceptual image patch similarity (LPIPS)^35^ were used for the latter.

##### PSNR

The PSNR is used as an index of image quality degradation in image compression. For a reference image *f* and a target image *g* both with sizes of *M* × *N*, the ratio of the square of the maximum pixel value that the image can encode (255 for 8-bit images) to the mean squared error (MSE) between the images is expressed in decibels (Eq. (4)). The higher the value is, the closer the quality of the reference image is maintained.

4 $$\begin{array}{c}PSNR\left(f,g\right)=10{\text{log}}_{10}\left(\frac{{255}^{2}}{MSE\left(f,g\right)}\right),\end{array}$$

5 $$\begin{array}{c}{\text{where}}\, MSE\left(f,g\right)=\frac{1}{MN}{\sum }_{i=1}^{M}{\sum }_{j=1}^{N}{\left({f}_{ij}-{g}_{ij}\right)}^{2}.\end{array}$$

##### SSIM

The SSIM focuses on three factors: image luminance, contrast, and structure. These factors are expressed in the mean pixel values *μ*_f_ and *μ*_*g*_, standard deviations *σ*_*f*_ and *σ*_*g*_ of images *f* and *g*, and covariance *σ*_*fg*_ of the two images (Eq. (6)).

6 $$\begin{array}{c}SSIM\left(f,g\right)=\frac{\left(2{\mu }_{f}{\mu }_{g}+{C}_{1}\right)\left(2{\sigma }_{fg}+{C}_{2}\right)}{\left({\mu }_{f}^{2}+{\mu }_{g}^{2}+{C}_{1}\right)\left({\sigma }_{f}^{2}+{\sigma }_{g}^{2}+{C}_{2}\right)}.\end{array}$$

Values are given in the range of 0–1, with a value closer to 1 indicating a better image quality. When calculating for 8-bit images, constant terms ${C}_{1}=0.01\times {\left(255\right)}^{2}, {C}_{2}=0.03\times {\left(255\right)}^{2}$ are generally used, and these values were also used in this study.

##### FID

The FID shows the similarity between two image groups as a distance. The features of 2048 dimensions obtained by the final pooling layer of Inception-v3^36^, a type of CNN, were extracted from all images belonging to image groups *F* and *G* for comparison. The FID was calculated using Eq. (7) with the average *µ*_*F*_ and *µ*_*G*_ of the extracted features in each image group and covariance matrices Σ_*F*_ and Σ_*G*_.

7 $$\begin{array}{c}FID\left(F,G\right)={\Vert {\mu }_{F}-{\mu }_{G}\Vert }_{2}^{2}+{\text{T}}{\text{r}}\left({\Sigma }_{F}+{\Sigma }_{G}-2\sqrt{{\Sigma }_{F}{\Sigma }_{G}}\right).\end{array}$$

Smaller values indicate a higher similarity between image groups. Pre-trained weights were applied to Inception-v3 using ImageNet, a large database of natural images^37^.

##### LPIPS

The LPIPS is a measure of the similarity between images, which was proposed based on the remarkable image recognition performance of CNN models to solve the problem that the results of conventional measures differ from human perception. The two images to be compared, *f* and *g*, were input to the CNN. From each image, a feature map ${\widehat{f}}^{l},{\widehat{g}}^{l}$ was obtained of size *M* × *N* from convolutional layer *l*. Similarity between the images was calculated by taking the pixel-by-pixel difference between all feature maps, weighting them with *w*, averaging them, and then summing them (Eq. (8)).

8 $$\begin{array}{c}LPIPS\left(f,g\right)={\sum }_{l}\frac{1}{{M}_{l}{N}_{l}}{\sum }_{M,N}{\left\Vert {w}_{l}\odot \left({\widehat{f}}_{MN}^{l}-{\widehat{g}}_{MN}^{l}\right)\right\Vert }_{2}^{2}.\end{array}$$

A smaller value indicates a higher similarity between images. AlexNet^38^, VGG^39^, and SqueezeNet^40^, which were trained on ImageNet, can be used to extract feature maps, and AlexNet was used in this study. The weights *w* were determined using a neural network comprising three fully connected layers. This network was pre-trained on the BAPPS dataset^35^, which is a specially designed dataset for calculating the LPIPS, and no additional training was conducted. The BAPPS dataset contains more than 160,000 sets of three images, including a reference image and two distorted images created via image processing. The dataset is designed for models to select an image that is more similar to the reference image among the two distorted images. The distorted images selected by human perceptual judgments were compared with those selected based on the calculation of the LPIPS, and the network was trained to obtain weights *w* such that the two images matched.

### Data augmentation using the generated images for a classification task

The proposed method is intended for use in data-augmentation applications. For a more practical evaluation, we verified the effectiveness of the images generated via data augmentation in a lung cancer histological classification task using CT images.

#### Dataset split for the classification

All lung cancer cases used in this study were pathologically examined, and histological types were determined for adenocarcinoma (ADC), squamous cell carcinoma (SCC), or small cell carcinoma (SCLC). There were 49 ADCs, 54 SCCs, and 44 SCLCs. Of these, 26 each, for a total of 78, were used as data for histological classification. These 78 tumors were identical to the data used in previous research^12^, which shared a part of the dataset. Of the remaining 69 images, 10 for each histological type, for a total of 30, were used as test data for image generation, and the remaining 39 were used as training data for image generation.

#### Experiment settings

Both pix2pix and StylePix2pix were trained using the same preprocessing and hyperparameters as in “Image preprocessing”, “Pix2pix”, and “StylePix2pix”. When generating images, edges extracted from the above 30 test data points were used after the number of images was increased by rotation (“Image preprocessing” section). For StylePix2pix, the input of the mapping network was randomly changed, and five images were generated from each edge. The total number of images generated was 980 images × 3 classes and 4900 images × 3 classes for pix2pix and StylePix2pix, respectively.

AlexNet, implemented in Tensorflow2, was used as the CNN for the classification. After pre-training all layers using generated images, only the fully connected layers were fine-tuned with real images. The pre-training phase had a learning rate of 1 × 10^–5^, 100 epochs, and batch size of 32, and the fine-tuning phase had a learning rate of 1 × 10^–4^, 100 epochs, and batch size of 8. Optimization algorithms were all stochastic gradient descent (SGD). Calculating the classification accuracy is also presented as the mean and standard deviation of three iterations of the threefold cross-validation method, as in prior research.

## Results

### Image generation using edges

**Figure 5:**
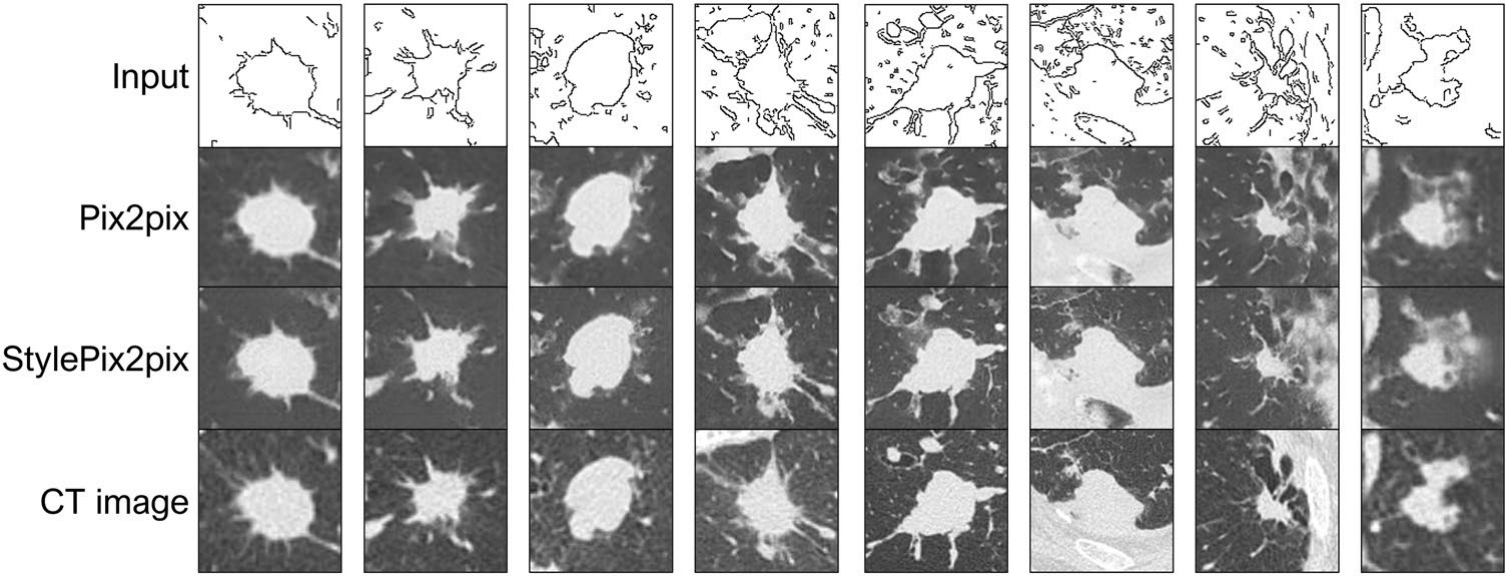
Examples of the image generation result using edges.

Figure 5 presents the results of edge-based image generation on the test data, wherein random numbers following a standard normal distribution were used as the input of the style blocks of the StylePix2pix model. While most lesions were precisely represented using either model, representing the surrounding blood vessels and structures in the chest wall was difficult, and many showed artifacts.

Table 1 lists the calculation results for the four metrics. Only the FID was calculated using all 20 test images for comparison between image groups, and the remaining images were the average values of the comparison for each image. StylePix2pix showed better results for all metrics.

**Table 1:** Quantitative evaluation of generated images from edges.

	Metrics			
	Higher is better		Lower is better	
	PSNR	SSIM	FID	LPIPS
Pix2pix	17.92	0.675	251.1	0.260
StylePix2pix	**18.73**	**0.682**	**220.1**	**0.225**

Significant values are in bold.

### Image generation using hand-drawn sketches

**Figure 6:**
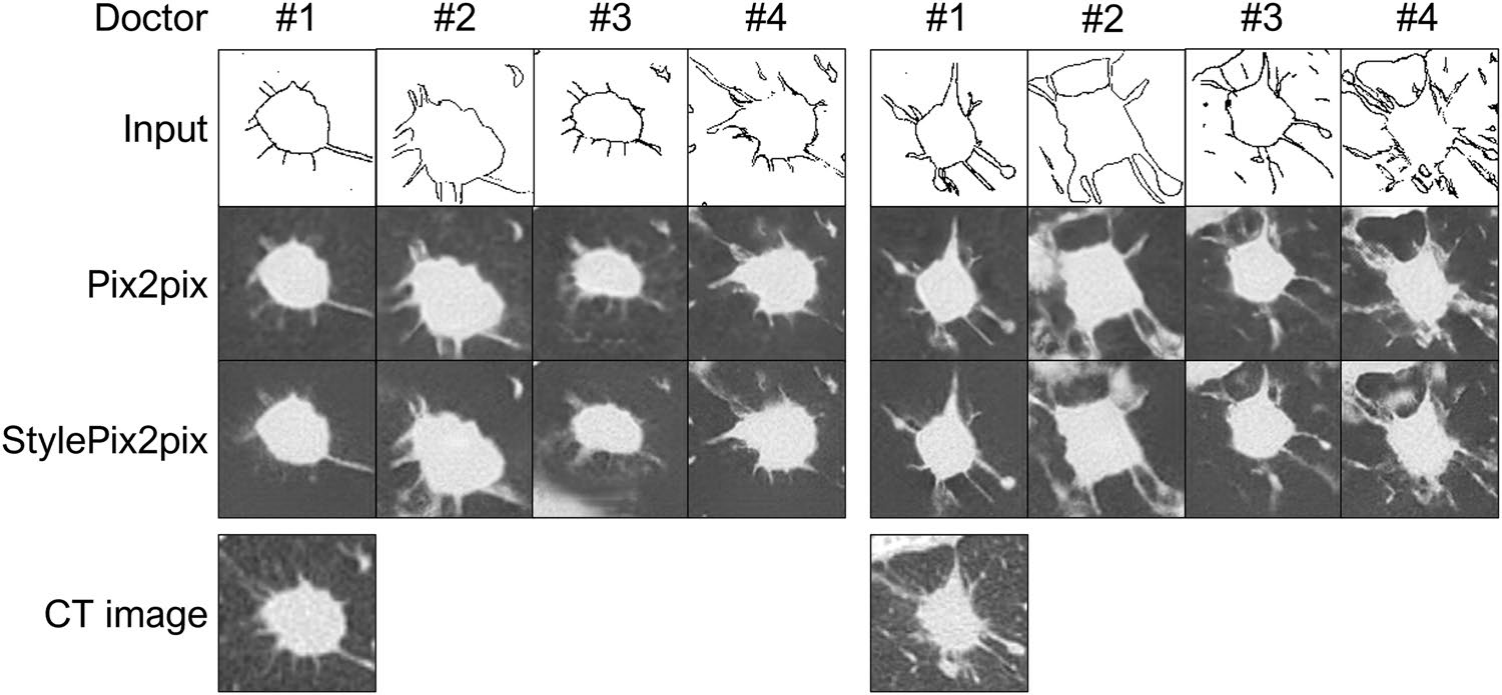
Examples of the sketches and image generation result using edges.

Figure 6 presents the sketches of each of the four doctors and results of the image generation based on them, where the input of StylePix2pix was random, as in the case of edges. Doctor #1 drew only the lesion, whereas Doctors #2 and #3 drew structures that could be recognized relatively clearly on the CT image in addition to the lesion. Doctor #4 drew detailed microstructures. In all cases, lesions were precisely represented, and the surrounding structures were either incompletely represented or accompanied by artifacts, as was the case when edges were used. Table 2 lists the calculation results of the metrics when sketches are used. The calculation method was the same as that used for edges. Conversely, the model exhibiting better values varied depending on both the metric considered and the doctor who drew the sketch.

**Table 2:** Quantitative evaluation of generated images from sketches.

	Doctor			
	#1	#2	#3	#4
**(a) PSNR**				
Pix2pix	12.51	13.90	**13.65**	14.80
StylePix2pix	**12.52**	**13.97**	13.41	**14.97**
**(b) SSIM**				
Pix2pix	0.5001	0.4826	**0.4769**	**0.5036**
StylePix2pix	**0.5052**	**0.4882**	0.4690	0.4990
**(c) FID**				
Pix2pix	**292.8**	287.4	294.2	281.5
StylePix2pix	322.2	**280.7**	**284.9**	**266.5**
**(d) LPIPS**				
Pix2pix	**0.4666**	0.3951	**0.3877**	0.3170
StylePix2pix	0.4841	**0.3867**	0.3991	**0.3123**

Significant values are in bold.

**Figure 7:**
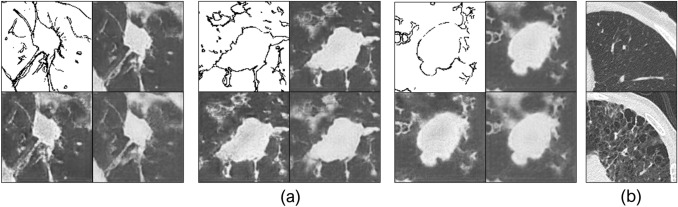
Results of image generation varying input of the style block. (**a**) Examples of generated images. (**b**) Comparison of lung substances in the dataset (upper: healthy, lower: pneumonia).

We also investigated the effect of changing the input of the style block on the image, and Fig. 7a presents the results. Generate multiple images from a single sketch is possible.

### Result of data augmentation application

Table 3 presents the results of the application to lung cancer classification. The classification accuracy when using only real and InfoGAN-generated images were taken from prior research^12^ for comparison. Improvement in classification accuracy was confirmed using the generated images in both cases of pix2pix and StylePix2pix.

**Table 3:** Classification results.

Generated images	Classification accuracy [%]
None^12^	34.2 ± 5.1
InfoGAN (10,000 × 3 classes)^12^	57.7 ± 5.3
Pix2pix (980 × 3 classes)	40.3 ± 5.0
StylePix2pix (4900 × 3 classes)	48.7 ± 3.1

## Discussion

As the lesion was precisely represented regardless of the presence or absence of style blocks, edges, or sketches, the U-net structure may have accurately transferred the shape and location of the sketch. However, as the main input information comprised only sketches, distinguishing between the lesion and others was impossible, which may be the reason for the difficulty in representing the surrounding structures. SPADE, a technique derived from GAN, can generate complex landscape images by inputting color-coded representations of landscape elements (e.g., sky, trees, and houses)^41^. Following this method, this problem can be solved by distinguishing the lesion, chest wall, mediastinum, and so on, by color.

As the values of all metrics in Table 1 were better, StylePix2pix may have been able to output images with high similarity to real images and good image quality when edges were used. Conversely, when sketches were used, the PSNR and SSIM values were almost the same for both models (Table 2). The results of FID and LPIPS were better when using Pix2pix for coarse sketches, such as those of Doctor #1 and, conversely, when using StylePix2pix for fine sketches, such as with Doctor #4. Although the edges used for training varied from fine for large tumors to coarse for small nodules, learning and representing them comprehensively in either model was impossible. In the case of StylePix2pix, unnatural streak artifacts were sometimes observed in the blank area without drawing, particularly around the lower bound of the image, suggesting that the model may have learned to recognize only the area near the drawn lines as the style. Skip connections in the U-Net structure are considered as involved in this problem; therefore, it may be solved in future work by changing the layer to apply the style block or by applying additional style blocks to the U-Net encoder. Additionally, cases wherein the edges detected by Canny edge detection showed apparent visual differences from sketches drawn by doctors. Recent edge detection methods, such as holistically nested edge detection^42^, have been demonstrated to extract more natural edges. Hence, application of such methods is expected to improve both models’ performance.

In feasibly generating images from coarse sketches, a difference in image quality of up to 35% was observed between sketches of Doctor #1, which completely excluded the surrounding structures, and those of Doctor #4, which were finely drawn. However, when comparing Doctors #2, #3 with practical levels of sketching and #4, most metrics had differences of approximately 10% and were, therefore, considered acceptable. Thus, image generation can be possible without major problems, unless the sketch is highly omitted.

Most of changes occurring in the output image owing to the adoption of the style block were related to image quality (e.g., contrast and sharpness). In this study, we used data obtained from a single imaging facility and equipment, and imaging conditions were basically the same; therefore, imaging environment being a factor is unlikely. In contrast, our dataset included patients with severely damaged lungs owing to complications such as pneumonia (Fig. 7b). Images of such patients tend to be degraded owing to changes in the X-ray absorption in the lung fields. Therefore, these differences in image quality under such bad conditions were acquired as styles and represented in the generated images. Although the current expressions of StylePix2pix remaining within the range are replaceable by conventional image processing, StylePix2pix can output more diverse images if trained by adding images taken under more widely varying environments and conditions.

Results of the data augmentation application showed that the classification accuracy improved, although not as much as that with InfoGAN in prior research. InfoGAN is specialized for this task, such as explicitly providing information on each histological type as input to generate images. In contrast, the proposed methods improved in the classification accuracies without including any processing designed for this task. Therefore, the feasibility of general-purpose data augmentation using complex shape representation is suggested. Furthermore, StylePix2pix was more effective in improving the accuracy than pix2pix, indicating the benefit of large-scale data generation.

In this study, a private dataset was used for the analysis. Additional experiments using publicly available datasets, such as LUNA16^43^, should be conducted in the future to compare this method with other GAN-based lung nodule image-generation methods. Additionally, as none of the quantitative metrics employed in this study can fully express the reality of the generated images, additional verification is necessary in the future as a more suitable metric is established.

## Conclusion

In this study, as an initial investigation for generating desired shaped lesion images, we aimed to generate lung cancer CT images based on sketches using pix2pix, an image-to-image translation technology. Additionally, we proposed StylePix2pix as an improved version of pix2pix, designed to increase its applicability in data augmentation. Although the representation of surrounding structures can still be improved, both models can represent tumors with complex shapes, indicating the feasibility of generating lesion images with arbitrary shapes. Additionally, StylePix2pix could acquire multiple images from a single sketch, suggesting its applicability to data augmentation.

## Data Availability

The datasets analyzed in this study are not publicly available because they include patient information. The source code used in this study is available from the corresponding author upon reasonable request.
